# Crosstalk Suppressed 3D Light Field Display Based on an Optimized Holographic Function Screen

**DOI:** 10.3390/mi13122106

**Published:** 2022-11-29

**Authors:** Hui Zhang, Xunbo Yu, Xin Gao, Chongli Zhong, Yingying Chen, Xinzhu Sang, Kuiru Wang

**Affiliations:** State Key Laboratory of Information Photonics and Optical Communications, Beijing University of Posts and Telecommunications (BUPT), Beijing 100876, China

**Keywords:** 3D light field display, holographic function screen, crosstalk suppression, backward ray tracing

## Abstract

A holographic function screen (HFS) can recompose the wavefront and re-modulate the light-field distribution from a three-dimensional (3D) light field display (LFD) system. However, the spread function of existing HFSs does not particularly suit integral imaging (II) 3D LFD systems, which causes crosstalk and reduces the sharpness of reconstructed 3D images. An optimized holographic function screen with a flat-top rectangular spread function (FRSF) was designed for an II 3D LFD system. A simulation was carried out through ray tracing, which verified that the proposed diffusion function could suppress crosstalk and improve the overall effect.

## 1. Introduction

Three-dimensional (3D) light field display (LFD) aims to provide vivid and natural scenes and nature-like 3D simulation environments, and is considered a promising method to reconstruct the light rays’ distribution of a real 3D scene [1,2,3,4,5]. There are many types of 3D LFDs, such as holographic displays [6,7], 3D LFD displays based on a parallax barrier [8,9] or lenticular lens sheet [10,11,12], auto-stereoscopic 3D LFDs based on a projector array [13,14,15], and integral imaging (II) based on a pinhole array or microlens array (MLA) [16,17,18,19]. MLA-based II has been extensively investigated in recent years, through which a 3D description of the natural light field with continuous horizontal and vertical motion parallax can be described [20,21,22]. Through the refraction of a MLA, the position and color information of different pixels are transformed into the direction information of light intensity of different viewpoints; the purpose of reconstructing the light field of 3D objects is realized using II. In a conventional II system, the observer naturally focuses on the lens array instead of the 3D images, resulting in visual discomfort. In addition, the gap between the adjacent lenses in the lens array results in a brightness discontinuity of the 3D images, which detracts from the visual experience.

With the rapid advances in optoelectronic, electronic, and computer processing technologies, novel components and processing techniques can be used in 3D displays. A holographic function screen (HFS) is a special holographic optical element that combines diffraction and diffusion, which can eliminate the gap between the lenses to guarantee the continuity and uniformity of a reconstructed 3D image. The modulation function and realization method of a HFS are detailed in the literature [23,24,25]. To improve display performance, a HFS has been added to a display system and achieved the desired results. X. Sang et al., proposed a 3D display system with a special HFS that can display a fully continuous, natural 3D scene with more than 1 m image depth in real time [26]. X. Yan et al., used a holographic diffuser to enhance the viewing resolution of an II-based light field display [27]. Afterward, X. Sang, et al., applied a HFS to a MLA-based II and achieved a clear and natural 3D light field image without the influence of the pattern of the lens array [28]. Through this research, the importance of HFSs in the field of 3D LFD was verified. However, a conventional HFS with a circular spread function (CSF) does not particularly suit an II 3D LFD (hereafter referred to as a 3D LFD), because the structure of a 3D LFD system and the spread function do not exactly match.

In this study, a HFS with a flat-top rectangular spread function (FRSF) was designed for a 3D LFD, and the overall display effect of the 3D LFD system was simulated by backward ray tracing (BRT) to aid in the evaluation of the proposed HFS. The HFS’ display effects with the conventional spread function and proposed spread function were compared and the suppression of crosstalk and improvement in the overall display effect of the proposed spread function were verified.

## 2. Basic Principle

### 2.1. Architecture of the 3D LFD

**Figure 1:**
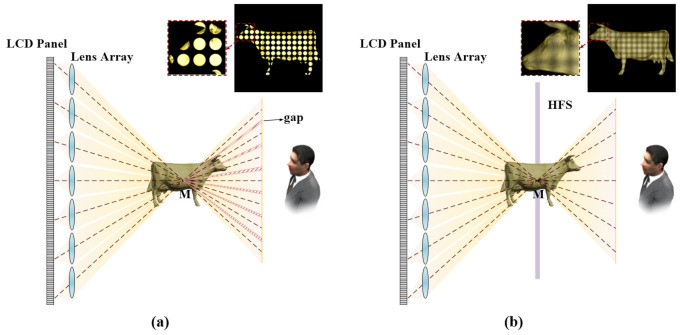
The schematic diagram (**a**) without HFS, and (**b**) with HFS.

The schematic diagram of the original 3D LFD system is shown in Figure 1a. The elemental image array (EIA) was loaded by the LCD panel. The ray generated by subpixels on the LCD panel passed through the corresponding microlens, converged at the center point M of the reconstructed object, and was then directed into human eyes. The entire optical system consisted of LCD and a MLA. In this structure, the resolution of the reconstructed image that human eyes can perceive was the number of microlenses; when human eyes focus on the lens array for a long time, it creates visual discomfort. Moreover, the distance between adjacent lenses will lead to the discontinuity of the 3D image and the non-uniformity of brightness, as indicated in the shadow areas in Figure 1a.

Therefore, a HFS was introduced into an LFD system. It has been proven that a HFS can modulate the input light field to optimize the display effect. A HFS was placed on the image plane of a MLA, and human eyes focused on the HFS, which greatly reduced viewing discomfort. After being modulated by the HFS, the light incident on the HFS emitted a beam with a certain diffusion angle, which was used to reconstruct the light distribution from the MLA to approximate the light field distribution of the real 3D scene. Through the diffusion effect of the HFS, the effect of the gap on the microlens array to the 3D image was also smoothed. Compared with Figure 1a, the light rays that converged at the point M did not go directly into the human eyes, but were scattered by the HSF, as shown in Figure 1b. The whole optical system consisted of the LCD plane, a MLA, and a HFS. The 3D scene reconstructed by this system had a weak sense of fragmentation and better continuity.

### 2.2. Modulation Function of the Holographic Function Screen

**Figure 2:**
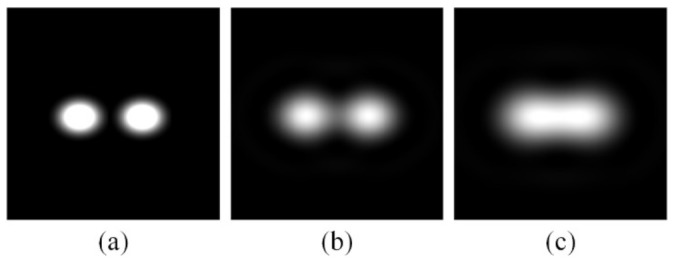
Diffusion effects (**a**) without a HFS, (**b**) of a HFS with a 1.5° diffusion angle, and (**c**) of a HFS with a 3° diffusion angle.

A holographic function screen (HFS) is a special holographic optical element combining diffraction and diffusion. Figure 2 illustrates the effect of HFS with different diffusion angle parameters; each spread function was a circular spread function (CSF). Figure 2b,c show the diffusion effect of a 1.5° diffusion angle and a 3° diffusion angle, respectively. A HFS with different parameters has been widely used in 3D field displays. When a HFS is illuminated by light refracted by the microlens array, the light field distribution is re-modulated. The HFS synthesizes the fragmented images, which is the key to realizing a high-quality 3D display.

**Figure 3:**
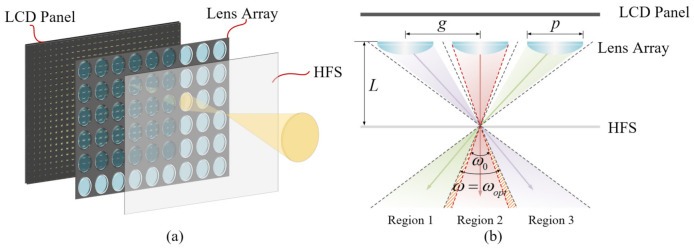
Schematic diagrams of the display principle: (**a**) the light modulation process using the lens array and the HFS; and (**b**) the relationship between the diffusion angle of the HFS and the lens parameters.

A HFS can diffuse the incident light at a fixed diffusion angle. In the 3D LFD system, a HFS was placed on the image plane of the microlens array. The light emitted from each lens at different angles diffused into the vertebral body through the HFS, as shown in Figure 3a. The HFS diffused the incident light at a certain angle so that the light emitted by the microlens array could fully diffuse into the whole field of view space, allowing the observer to see a continuous image. The diffusion angle was related to the specific parameters of the LFD system. As shown in Figure 3b, L is the distance between the lens array and the HFS, p is the aperture of the lens unit, and g is the distance between the centers of the two adjacent lenses. $\omega_{0}$ is the light beam angle without the diffusion of the HFS. The reconstructed image could not be observed in the two shaded areas without the diffusion of the HFS; therefore, the reconstructed image was discontinuous. ω is the light beam angle after the diffusion of the HFS. Theoretically, the optimal diffusion angle $\alpha_{opt}$ can achieve no gap or overlap between the three regions [29]; the optimal light beam angle $\omega_{opt}$ and the optimal diffusion angle $\alpha_{opt}$ can be calculated using the following equations:

(1) $$\omega_{opt}=2\times \arctan(\frac{g-p}{2L})$$

(2) $$\alpha_{opt}=\omega_{opt}-\omega_{0}$$

## 3. Analysis and Redesign of the HFS Spread Function

**Figure 4:**
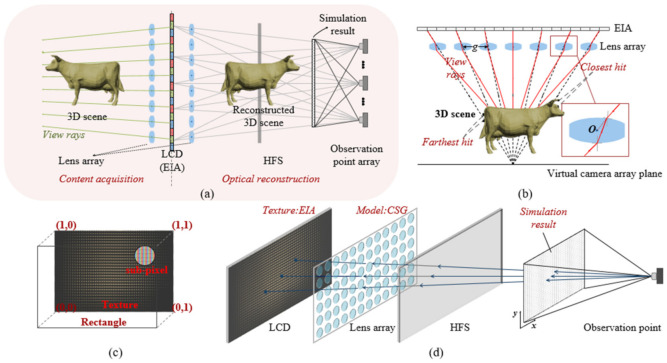
Schematic diagrams of the simulation system: (**a**) the simulation process of content acquisition and optical reconstruction; (**b**) the EIA generation process based on BRT; (**c**) the texture of the LCD plane model; and (**d**) the reconstructed result computation process based on BRT.

The 3D LFD system based on HFS with a conventional spread function was simulated. OptiX 6.5 was used as the ray tracing engine, and the whole simulation system was divided into two parts, content acquisition and optical reconstruction, as shown in Figure 4a [30]. The elemental image array (EIA) was obtained through a content acquisition process, and then loaded onto the LCD panel as a texture, as shown in Figure 4b,c. In the optical reconstruction process, the reconstructed results were obtained based on the BRT, as shown in Figure 4d.

**Figure 5:**
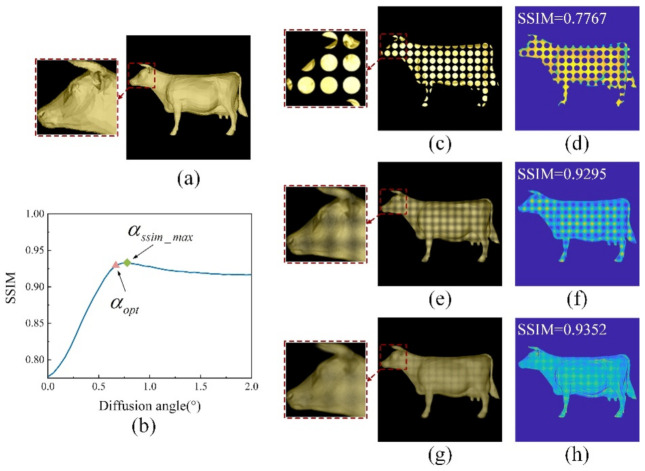
Simulations of different diffusion angles of the 3D scene: (**a**) image of the cow model taken directly by the virtual camera; (**b**) simulation of the relationship between the diffusion angle of the HFS and the SSIM value; (**c**) simulated reconstructed result without the HFS; (**d**) SSIM corresponding to the reconstructed result in (**c**); (**e**) simulated reconstructed result using the HFS with the optimal diffusion angle $\alpha_{opt}$; (**f**) SSIM corresponding to the reconstructed result in (**e**); (**g**) simulated reconstructed result using the HFS with the max SSIM value diffusion angle $\alpha_{ssim\_max}$; and (**h**) SSIM corresponding to the reconstructed result in (**g**).

The simulation results are shown in Figure 5. Figure 5a is an image of the model taken directly by the camera. Figure 5b shows the relationship between the value of SSIM and the diffusion angle of the HFS obtained via simulation. As the diffusion angle of HFS increased, the value of SSIM first increased, and then decreased. Theoretically, a reconstructed image at the optimal diffusion angle has no crosstalk and no gaps, and the optimal diffusion angle corresponds to the best-reconstructed image. However, the simulation results in Figure 5b show that the SSIM value of the reconstructed image that corresponded to the optimal diffusion angle $\alpha_{opt}$ was not the highest. Figure 5e is the reconstructed result when the diffusion angle was $\alpha_{opt}$, and Figure 5f is the SSIM diagram corresponding to Figure 5e. Figure 5g is the reconstructed result when the diffusion angle α($\alpha =\alpha_{ssim\_\max}$) was greater than $\alpha_{opt}$, and Figure 5h is the SSIM diagram corresponding to Figure 5g. Comparing Figure 5e,f and Figure 5g,h, it can be seen that when $\alpha =\alpha_{ssim\_\max}$, the reconstructed result was better than when $\alpha =\alpha_{opt}$, and the SSIM value was also higher. However, comparing the magnified sections of Figure 5e,g, it can be seen that although the light intensity of Figure 5g was smoother, the details were blurred.

**Figure 6:**
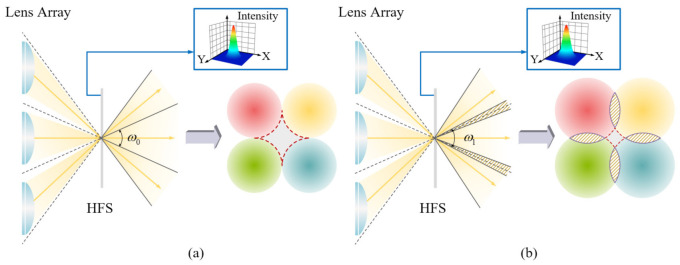
Different spread effects corresponding to different diffusion angles: (**a**) the spread effect corresponding to the optimal diffusion angle; and (**b**) the spread effect corresponding to moderate over diffusion.

As shown in Figure 6, when $\alpha =\alpha_{opt}$($\omega =\omega_{opt}$), although there was no overlap because the spread function of HFS was CSF, there was still a large gap in the middle, which made the image’s continuity inadequate. When the diffusion angle was increased, although there was some aliasing between the images, the missing information decreased and the images were more continuous, so the SSIM value increased instead. However, when the diffusion angle continued to increase, the effect of crosstalk caused by aliasing on the reconstructed image was greater than the reconstructed image’s quality improvement due to filling in missing information. The reconstructed image became blurred and the SSIM decreased.

**Figure 7:**
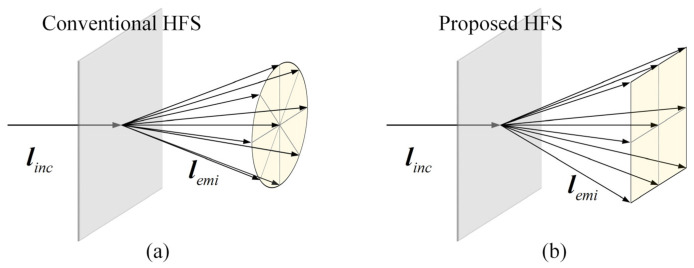
Emitted light distribution with (**a**) conventional HFS, and (**b**) proposed HFS.

If the spread function of HFS was changed to reduce the amount of missing information while maintaining the non-aliasing of the reconstructed images, the continuity of the image could be guaranteed and the crosstalk could be suppressed without causing image blur. Therefore, a flat-top rectangular spread function was designed, and a geometric model of the HFS with the rectangular flat-top spread function in the simulation system was established. The closest hit functions of the HFS in the simulation system were changed to achieve different diffusion effects, as shown in Figure 7. When the incident light hit the HFS, the emitted light distribution was different.

**Figure 8:**
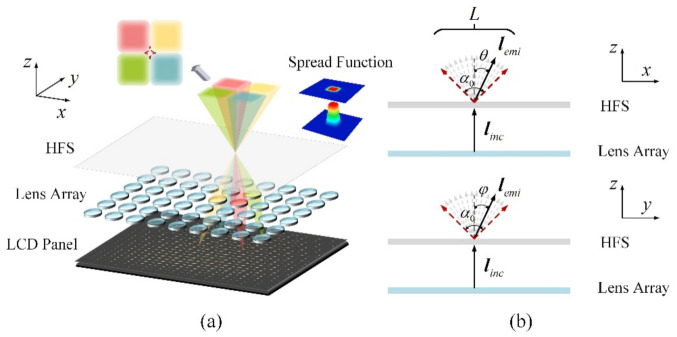
(**a**) Schematic of the 3D LFD system based on the HFS with the proposed spread function, and (**b**) cross-sectional views of the light diffusing process.

The schematic diagram of the simulation system is shown in Figure 8. When the incident light ray was on the HFS, the light beam intensity was uniformly diffused at angle $\alpha_{0}$ in the horizontal and vertical directions. The collection of the light rays emitted in different directions can be expressed as:

(3) $$L=\left\{l_{emi}|lL_{emi}=R(i,\theta )\cdot R(k,\varphi )\cdot l_{inc},\theta \in \left(-\alpha_{0}/2,\alpha_{0}/2\right),\varphi \in \left(-\alpha_{0}/2,\alpha_{0}/2\right)\right\},$$

where i and k are the unit vectors of the coordinate axes, and $l_{emi}$ and $l_{inc}$ are the emitted light ray vector and the incident light ray vector, respectively. R(x,θ) and R(z,φ) in Equation (3) are rotation matrixes that can make the incident light ray rotate θ degree around vector i and rotate φ degree around vector k.

## 4. Experimental and Simulation Results

**Figure 9:**
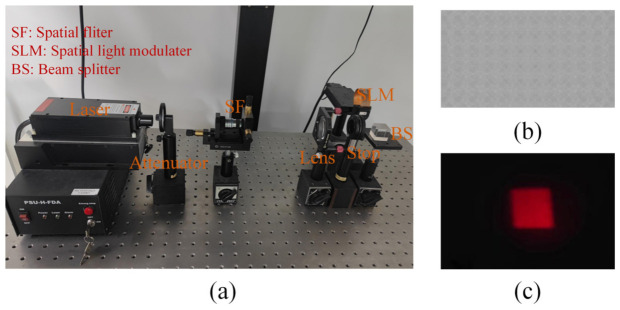
(**a**) The optical experiment setup; (**b**) the CGH; and (**c**) the experimental result.

A verification experiment was conducted to demonstrate the flat-top rectangular diffusion effect of the proposed HFS. In the experiment, a spatial light modulator (SLM) loaded the computer-generated hologram (CGH), and a laser beam illuminated the SLM. A rectangular light spot was obtained on the receiving screen, as shown in Figure 9.

The parameters and values used in the simulations are provided in Table 1, and the relative position of each component is shown in Figure 1b. The simulations and optimizations were carried out using a PC with an Intel(R) Core (TM) i7-4790 CPU @ 3.6 GHZ, 8 GB RAM, and a NVidia GeForce GTX 1660Ti (4 GB/NVidia) graphics card. The simulation program was implemented via CUDA SDK 8.0 and OpenGL4.0.

**Figure 10:**
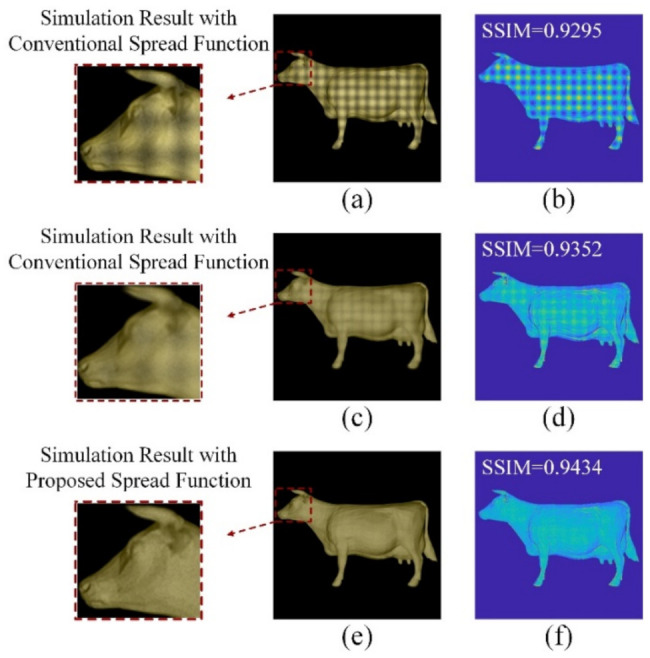
Simulation results comparison: (**a**) simulated reconstructed result with the conventional spread function at the optimal diffusion angle; (**b**) SSIM corresponding to the reconstructed result in (**a**); (**c**) simulated reconstruction result with the conventional spread function at the highest value of SSIM; (**d**) SSIM corresponding to the reconstructed result in (**c**); (**e**) simulated reconstruction result with the proposed spread function at the optimal diffusion angle (also the highest value of SSIM); and (**f**) SSIM corresponding to the reconstructed result in (**e**).

The simulated results using the conventional spread function and the proposed spread function are shown in Figure 10. Figure 10a,c show that the continuity of the reconstructed image had a more obvious impact on visual perception than clarity. Therefore, to obtain a better 3D visual experience, in the design of HFS with conventional spread function, the diffusion angle was appropriately increased to improve the continuity of the image. However, this led to increased crosstalk and decreased image clarity. Comparing Figure 10a,e, the clarity of simulated results was similar, but it is obvious that the simulated result in Figure 10e had better continuity, and the SSIM value was higher. Comparing Figure 10c,e, the continuity of simulated results was similar, but the simulated result in Figure 10e had better clarity and a higher SSIM value. That is to say, the HFS with the proposed spread function suppressed crosstalk while ensuring the continuity of the reconstructed image.

**Figure 11:**
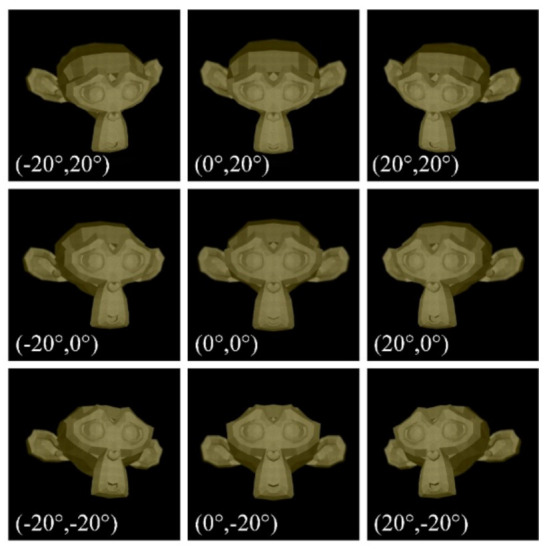
Simulation results of the reconstructed monkey head captured from 3 × 3 positions.

The 3D LFD display results of reconstructed images (the monkey head) observed from different directions are presented in Figure 11, showing that clear and continuous 3D images could be perceived.

## 5. Conclusions

In summary, to effectively suppress crosstalk and improve the display effect of the 3D LFD system, the spread function of the HFS was analyzed and redesigned. An HFS with a flat-top rectangular spread function (FRSF) was designed. Simulations based on BRT were used to simulate and evaluate the display effect of the 3D LFD system. The SSIM value of the reconstructed 3D image using the redesigned HFS was higher than that reconstructed using the conventional HFS; crosstalk was suppressed and the quality of the reconstructed images was significantly better than those of the conventional HFS, which proved the effectiveness of the proposed HFS.

## Figures and Tables

**Table 1:** The main parameters of the simulated 3D LFD system configurations.

Parameters	Values	Unit
Lens gap (g)	11.28	mm
Pitch of lens (p)	8.60	mm
Focus of lens (f)	19.55	mm
Distance between the lens array and the HFS (L)	230.10	mm
Diffusing angle of the HFS ($\alpha_{opt}$)	0.67	°
